# ‘A life in a day’ simulation experience: perceptions of oncology nurses and pharmacy staff

**DOI:** 10.1186/s12912-022-01086-8

**Published:** 2022-11-08

**Authors:** Rachel Ehibhatiomhan, Emma Foreman, Lisa Barrott, Jessica Shek, Shereen Nabhani-Gebara

**Affiliations:** 1 British Oncology Pharmacy Association, Kingston University, London, UK; 2 British Oncology Pharmacy Association, The Royal Marsden NHS Foundation Trust, London, UK; 3 UK Oncology Nursing Society, University Hospitals Sussex NHS Foundation Trust, Brighton, UK; 4 British Oncology Pharmacy Association, East Sussex Healthcare NHS Trust, Seaford, UK

**Keywords:** Patient simulation, Oncology nursing, Renal cell carcinoma, Empathy

## Abstract

**Background:**

Due to an increase in patient numbers, more cancer patients are being reviewed by non-medical healthcare professionals (HCPs), and it is essential that they can empathise with patients and care for them holistically. ‘A Life in a Day’ is a role reversal simulation (RRS) which demonstrates the challenges, choices and impacts that cancer patients face every day, facilitated by a Smartphone application (app). This study focused on renal cell carcinoma (RCC) and was designed to evaluate the impact of RRS on participants from the British Oncology Pharmacy Association (BOPA) and the UK Oncology Nursing Society (UKONS), and identify any changes made to clinical practice as a result.

**Method:**

A survey was conducted via the app before and after the experience. Individual semi-structured interviews were conducted with participants over Microsoft Teams.

**Results:**

Data from the survey showed that after the experience 97% of all participants strongly agreed that they ‘feel empathy for RCC patients’ and 90% strongly agreed that they ‘feel inspired to place patients at the centre of their work’. There were 5 themes extrapolated from the qualitative data: Holistic understanding of Patients, Reflections on Practice, Changes in Practice, Outreach to Colleagues, Education & Training.

**Conclusion:**

Participants reported an increase in empathy for their patients which inspired them to make changes to their practice. This involved being more holistic in their care and taking on more responsibility. They recommended use of RRS for HCP training and continued professional development. They also suggested incorporation of RRS into the pharmacy undergraduate curriculum.

## Introduction

The incidence of cancer is on the rise, and patients are living longer with the consequences of cancer and its treatment [1]. This will result in up to 30% of cancer survivors experiencing one or more of; depression, anxiety, fatigue, pain, mood changes, cognitive dysfunction, and loss of control over daily activities [1]. As a result, the quality of life for cancer survivors is often reduced. Healthcare Professionals (HCPs) within the multidisciplinary team (MDT) are increasingly involved in supporting and caring for patients through their cancer journeys. It is unclear if traditional methods of pharmacy and nursing education have evolved to cater for this change. It is very important for HCPs to be empathetic and adequately trained to holistically care for their cancer patients. Pharmacy oncology training and education is currently heavily focused on the management of the disease rather than the individual, with insufficient in-person experience to facilitate smooth transition into practice. Simulation can be used to bridge this gap and adequately prepare HCPs for the changes in oncology practice. However, current training and education within oncology does not incorporate enough, if any, simulation and is therefore not providing adequate exposure to practical elements of oncology care [2].

The adult learning theory, developed by Malcolm Knowles, states that adults learn through experience, problem solving and receiving information relevant to their job or personal life [3]. Through simulation, learners are provided with the opportunity to learn in this format by being allowed to make mistakes in a safe environment. Simulation education is an innovative technique that can be used to enhance skills necessary for practice. This is not limited to technical skills such as surgical procedures, problem solving and decision-making skills, it can also support interpersonal skills such as communication and teamworking [4].

## Background

There are various ways in which simulation is used within training and education in healthcare, some include human patient simulation, virtual human/patient simulation, the use of mannequins, and more. A study was conducted in the U.S. where 206 second year medical students practiced communication skills using virtual human (VH) based simulation and an objective structured clinical examination (OSCE) [now5(was4)]. Students were asked to complete a reflective essay summarising their thoughts about both experiences with regards to understanding verbal communication and human interactions, amongst others. Identified themes included Learning Awareness of Nonverbal Skills, Gaining Useful Communication Skills suggesting an overall beneficial experience with both interventions. However, on comparison between VH and OSCE simulation it was mentioned that “Your true response can only come from human to human interaction…program is much stronger at allowing a person to think about their verbal responses” [5].

In a simulation study, standardised patients were used to train medical students in breaking bad news. The sample size consisted of 28 medical students and the control group included 38 medical residents. The intervention was carried out in the style of OSCEs with standardised breast and colon cancer patients [6]. As expected, results showed that residents were significantly better at showing rapport to standardised patients than students were (*p* = 0.015) [6]. However, there was no significant difference between the actual breaking of bad news or communication related to patients’ emotions between the two groups (*p* = 0.100, *p* = 0.828 respectively) [6]. The fact that students and residents showed no significant difference in communication skills relating to patient’s emotions, suggests that using standardised patients can be just as effective as having in-person experience with cancer patients.

Saiva et al. [7], carried out an immersive simulation intervention with a convenience sample of 15 medical residents at a Canadian mental health Hospital [7]. The aim was to provide medical residents insight into the struggles of geriatric patients. This was carried out by giving participants a suit to wear to emulate physical restrictions, ear plugs which played an audio to simulate hallucinations and difficulty in hearing. Goggles and gloves were worn to simulate dexterity problems and difficulty in seeing. During the simulation, participants were patients invited to a consultation with a pharmacist about their medicines and starting the use of Dossett boxes. Participants reported struggling to understand and hear everything that was said and felt limited as to how much they could engage and be a part of the consultation [7]. The voices in the audio caused great distress and anxiety which made the participants self-conscious. Before and after participating, residents completed a 7-point Likert scale test, which used the Jefferson scale of empathy to measure and compare levels of empathy before and after the experience. With 7 being extremely empathetic, both mean scores were compared, showing that empathy was significantly increased after the experience, *p* = 0.02, t = 2.65 [7]. A similar approach was used at the Department of Physical Medicine and Rehabilitation, Taipei Municipal Wanfang Hospital, China [8]. Thirty Physiotherapist interns participated in a simulation experience which involved completing tasks whilst wearing suits, to understand the struggles faced by older adults and those with disabilities [8]. Some tasks included; putting on and taking off clothes while wearing a hemiplegia simulation suit, using the nondominant hand to pick up beans with chopsticks, and drinking water while wearing a simulation suit. Interns completed tests before and after the intervention, which measured their empathy, knowledge, and attitude with the use of various scales. Results showed that interns had more empathy (*p* = 0.001), knowledge (*p* = 0.005), improved attitudes (*p* = 0.002) toward older adults and individuals with disabilities after the intervention [8].

Usually the majority of simulation education research is focused on the learner simulating the role of a future HCP. However, being put in the position of a patient through role reversal simulation (RRS), has rarely been done. Simulation of this nature is of growing interest as learners can better relate to the thoughts and emotions of patients, as well as have a better perception of how care is received. A Life in a Day is an application (app) created by The Method, a healthcare education company who uses theatre techniques to create realistic situations which elicit emotion and empathy [9]. It incorporates immersive RRS to teach HCPs about a particular patient group or chronic condition and what patients experience on a day-day basis [9]. This is a simulation experience where the individual becomes the patient for a day rather than simulating a future HCP role. Participants engage with stimuli in the form of app notifications and phone calls from actors who role play various characters. To create a heightened sense of reality, participants receive a kit with a range of items. Examples of these are red dye capsules to create the effect of haematuria when thrown into the toilet, or the wearing of a belt around the lower chest to simulate shortness of breath. The participant is required to carry on with their daily activities and work commitments whilst partaking in the experience to help participants appreciate the challenges faced by patients.

The ‘A Life in a day’ experience undertaken in this study was based on Renal Cell Carcinoma (RCC). RCC is the most common type of Kidney Cancer with a high 5-year survival rate of 70–80% [10]. Patients living with RCC can have different experiences, but many will experience fatigue, weight loss, haematuria, pain and a decrease in appetite [11].

### Aim

The aim of this study was to investigate the experience of pharmacy staff and nurses with the ‘A Life in a Day’ simulation experience.

## Method

This study adopted a mixed method approach where participants were invited to complete a survey and take part in a semi-structured interview. This study was carried out in collaboration with the British Oncology Pharmacy Association (BOPA), and the UK Oncology Nursing Society (UKONS). The inclusion criteria for this study were that participants had to be a registered nurse, pharmacist or part of the pharmacy team (such as pharmacy technicians, pharmaceutical sales representatives), a member of BOPA or UKONS and had participated in the RCC A Life in a day experience with The Method.

### Survey

A survey was conducted before and after the experience via the app, which invited all participants to rate their knowledge, confidence, empathy, and patient centricity on a Likert scale of 1–5, with 5 indicating ‘strongly agree’. Survey questions were created by The Method and were completed by participants via the ‘A Life in a Day’ app.

### Interview schedule

The interview schedule was split into 3 main sections: Personal impact, impact on practice, impact on others. The first section was focused on questions around the participant’s feelings, thoughts and expressions of the experience and any changes in attitudes towards patient care they may have noticed. Impact on practice, was the section based around the participant’s work tasks, and any changes that had been made or had been planned and how these would affect their patients. The last section explored if colleagues had been impacted in any way after being told about the simulation experience and what the participant’s thoughts were on their overall professional education and training and healthcare services provided. Prompts were included and used as and when necessary to encourage more elaborate responses from participants.

### Ethical approval

Ethical approval was obtained from Kingston University research ethics committee (KUREC) on 14th January 2021.

### Data collection

An initial pilot interview was held before participants were contacted. No changes to the interview schedule were made, as questions were well received. All participants were asked for informed written and verbal consent prior to interviews being held. Interviews lasted approximately 30 minutes over Microsoft Teams and were held from January 2021 to 31st March 2021. No sample size was calculated as interviews were held with the intention of collecting data until no new themes were identified and data saturation had been reached. To avoid bias or reduce the likelihood of missing new information, usually an extra 2 samples are taken at the point of data saturation [12]. In this case, data saturation was well exceeded.

A total of 38 HCPs, 23 pharmacy professionals and 15 registered nurses, took part in the experience. A cohort of 9 pharmacists piloted the experience in 2019 and 14 pharmacy staff carried out the experience in 2020. A cohort of 7 registered nurses completed the experience in September 2020 and a second cohort of 8 registered nurses completed the experience in November 2020. See Table 1 for a summary of participant profiles and Table 2 for a summary of interviews held.

**Table 1:** Summary of interviewed participants’ profiles

Participant	Profession	Work location	Job role
P1	Pharmacist	Industry	Medicine development
P2	Pharmacist	Hospital	Prostate and renal cancer
P3	Pharmacist	Hospital	Involved in clinical trials and oncology clinics (prostate & kidney)
P4	Pharmacist	Specialist cancer Hospital	Consultant Pharmacist (specialist area RCC)
P5	Pharmacist	Hospital	Chemotherapy day unit, training to become an IP^a^
P6	Pharmacist	Hospital	Split cancer clinical trials and inpatient oncology ward, training to become an IP
P7	Pharmacist	Hospital	Chemotherapy day unit
P8	Pharmacist	Hospital	Oncology&Haematology chemotherapy day unit
P9	Technician	Cancer private Hospital	Manager within pharmacy department, works with oncology pharmacists in chemotherapy unit
P10	Pharmacy team	Industry	Medicine sales and development
P11	Pharmacist	Hospital	Melanoma and renal cancer, IP
P12	Pharmacist	Hospital	Aseptic unit and involved in education & training
P13	Nurse	Hospital	CNS^b^ – metastatic prostate & renal cancer
P14	Nurse	Hospital	CNS - metastatic kidney cancer
P15	Nurse	Hospital	In charge of training oncology CNSs
P16	Nurse	Hospital	Clinics in Kidney and prostate cancer, involved in training & education
P17	Nurse	Hospital	CNS- Metastatic renal and prostate cancer
P18	Nurse	Hospital	Oncology inpatient ward
P19	Nurse	Hospital	Renal cancer

^a^Independent Prescriber, ^b^Clinical Nurse Specialist

**Table 2:** Summary of interviews held

	Pharmacy team	Registered Nurse	Total
Took part in experience	23	15	38
Consent to be contacted	15	12	27
Pilot Interview	1	0	1
Interview declined	1	2	3
No response/no interview scheduled	2	3	6
**Interviews held**	12 (incl pilot)	7	19

### Data analysis

All interviews were recorded, and saved on the interviewer’s Microsoft Streams secure account. All interviews were transcribed by RE verbatim on Microsoft Word. Transcripts were analysed through thematic analysis supported by NVivo 12. Thematic analysis identifies, organises, investigates, describes, and reports themes found within a data set [13]. This involved coding each transcript by highlighting and annotating relevant sections. Codes were carefully studied and put together into groups of commonalities. These were further grouped together into themes. Transcript coding and interpretation was a continuous and extensive process; this involved discussing and checking the coded transcripts with the lead author SNG. Any disagreements over data coding and interpretation were discussed until consensus was achieved. The final themes and subthemes were checked and verified by all authors to ensure validity of interpretations and consistency of the findings and to overcome bias in data analysis.

## Results

**Fig. 1:**
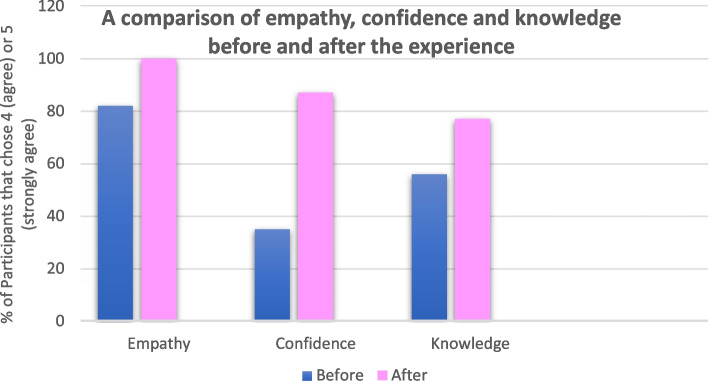
Participants were asked to complete a 5-point Likert scale survey (with 4 and 5 signifying ‘agree’ and ‘strongly agree’ respectively) before and after the experience. This figure shows the number of participants that agreed and strongly agreed with the questions based on their empathy for patients, confidence in practice and knowledge of renal cell carcinoma

Thirty four participants completed the 5 point Likert scale survey before and 30 participants after the experience. This allowed for a comparison providing insight into impact. See Fig. 1 for a comparison of empathy, confidence and knowledge before and after the simulation experience. 97% of participants agreed that the simulation experience helped them think about issues relating to the impact of cancer in a new way. Overall, 93% of all participants agreed that “A life in a day helped [them] increase the patient focus of [their] work”.

Fifteen individuals within the pharmacy team and 12 registered nurses gave their consent to be contacted for interviews. Of the 27 participants contacted, 1 nurse and 1 pharmacist declined doing the interview due to increased workload and 2 pharmacists and 4 nurses did not respond after several invites were sent or did not follow through in arranging for an interview date. One nurse responded after completion of data analysis, and this was declined. This resulted in a total sample size of 19 (including the pilot interview); 7 nurses, 10 pharmacists and 2 allied pharmacy staff. See Table 1 for a summary of participant profiles and Table 2 for a summary of interviews held.

### Outcomes – themes

After completion of analysis, 5 main themes were identified:

- Holistic understanding of the patient
- Reflections on practice
- Changes in practice
- Outreach to colleagues
- Education & Training.

#### Holistic understanding of the patient

Participants highlighted their feelings in response to the experience and for many, the experience gave participants insight into the emotions a cancer patient experiences when going through different stages in their journey. This was predominantly expressed by Pharmacy staff. Participants also felt that they gained a better understanding of the journey and various aspects a patient and their family go through in their daily lives when suffering from cancer. Some participants also had a better understanding of the disease and the patient’s experience of its impact, which increased participant empathy.

Feelings:

“I think one of the things that really stood out for me is, patients often do talk to us about something called scanxiety which is their anxiety they feel between waiting for the next scan and next treatment. That was definitely something I felt, although I was only waiting hours or minutes between the next part of the experience. I definitely felt anxious and checking my phone to see if anything had come through. So I feel like I could relate a bit more to that.”

P7, Pharmacist.

“When you’re talking to a patient, they don’t take half of what you’re saying because of the shock and that’s certainly something I experienced with this, especially when you get a message or phone call saying that you’ve got this diagnosis, you try to process that but they’re still talking, and you freeze the same way a patient would freeze and it was very enlightening to how the patient experience is.”

P12, Pharmacist.

Life & journey:

“It’s just understanding the patient journey and the impact it can have on their lives, because it’s not even just for the patients for their family as well…”.

P5, Pharmacist.

“…it does make me more empathetic to what’s going on and to what other situations people have going on in their lives outside the whole hospital system and the treatment system…”.

P12, Pharmacist.

“But I think it made me learn a lot more about renal cancer whereas before there were things, I didn’t have a clue, it made me learn and made me read a bit more around and understand a bit more.”

P17, Nurse.

#### Reflections on practice

Carrying out the experience prompted participants to reflect and share thoughts on their practice, approaches to patients and the services provided for patients. Participants had a positive outlook on healthcare services but noticed that many times there are other aspects of providing care that can be changed like spending more time with patients to engage in holistic consultation.

Reflections about self:

“…when that GP was saying about the diagnosis, she was so matter of fact and I’ve always taken that approach, that ‘let’s just tell it like it is, let’s just be upfront’. Actually, it’s quite harsh to speak to people like that and when she was telling me I was thinking ‘oh you’re doing what I do’, and I was listening to her thinking ‘I’m not really liking this’. It’s sort of funny to watch someone doing what you do yourself and then think oh okay perhaps that needs a bit of reflection.”

P14, Nurse.

Reflections about external factors:

“I think what patients need is somebody they can talk to that’s got the knowledge base but has also got time… I think it’s about that time to talk and really get to know people and not just, here’s your drugs, see you in 4 weeks’ time. I think it’s that sort of person that individuals need.”

P11, Pharmacist.

#### Changes in practice

Upon reflection, participants were inspired to change aspects of the care they provide. A recurrent concept was the increased empathy of participants, which sometimes motivated further changes in practice. Especially pharmacy participants were encouraged to interact more with their patients to be more visible and provide person centred care that was not just focused on medication.

Intrapersonal:

“When work is busy and pressure is quite high, it’s quite easy to see the patient as not too much as an individual but sort of another prescription to go through, because there’s another 10 prescriptions waiting for you but I think that the experience really changed that mindset to build the patient as an individual and they have their own life and family, jobs.”

P8, Pharmacist.

“The next day I had clinic and I was totally thinking about what I was saying, the language I was using, so immediately afterwards I was thinking how they feel, it really opened my eyes to give me a sense of what patients go through. It made me really choose my words carefully and allow I suppose more time for patients to respond, yeah definitely impacted me in practice.”

P16, Nurse.

Interpersonal:

“I think previously, especially for patients that are starting on their first cycle of chemotherapy, I would often let the nurses do the counselling on the medication but now I make an effort to do that myself. Then I feel like I am more visible as a pharmacist for them, they have my direct number if they have any medication queries. And I think that gives them a bit more reassurance that there’s people there…”.

P7, Pharmacist.

“But it has made me want to make sure that we do all we can to make sure that patient’s journey is as smooth as possible. So, for example, we have centralised aseptic units which supplies two different hospital sites so a 5 min delay in something can mean missing a transport slot which means a 3-hour delay to the patient at the other hospital site. So, you know is making sure that sort of thing does not happen or if it’s likely to happen, to get as much forewarning as possible so that the patient knows what to expect rather than getting rightfully wound up about it.”

P12, Pharmacist.

No change:

A few did mention not changing any aspects of their practice or the experience not having much of an impact on the practical elements of their practice. Some were not in patient facing roles and felt that that hindered them from making changes. Others felt that they did not need to make drastic changes due to their attained skills. However, more commonly participants felt that the experience had more of an impact on attitudes and perceptions which did not necessarily transfer to practice.

“It’s [practice] not changed as such because I am not physically seeing patients in front of me. If I was seeing patients in front of me, then you can see what they’re like, you can see their facial expression and you can delve further but now I am doing telephone calls so if the patient tells me something, I can’t do anything. So if we go back to patient facing appointments it can be easier.”

P11, Pharmacist.

“I wouldn’t say it’s necessarily changed the way that I approach patients, I would hope that I was already quite sensitive when I speak to them…”.

P14, Nurse.

“I’m not sure that I do things any differently, but I may just be a bit more conscious.”

P17, Nurse.

#### Outreach to colleagues

The experience of the role reversal simulation encouraged participants to share with colleagues and a wider audience via social media. Feedback from colleagues about the experience based on conversations was generally positive.

Informal discussion:

“I did speak with my colleagues, the other oncology pharmacists… it was quite interesting for them… They were intrigued…”.

P3, Pharmacist.

Formal discussion:

“I lead the cancer CNS meeting for the trust, and I actually put it on the agenda to talk about ‘cause I just thought it had such a big impact on me. I just wanted to share and just say to other people to, if given the opportunity, to absolutely get involved.”

P16, Nurse.

Promotion:

“I did quite a lot of talking with my colleagues and promoted it on Twitter as well just to make sure that people are aware…”.

P7, Pharmacist.

No discussion:

“Unfortunately, the time it occurred was the time this pandemic started kicking… our aseptic service kind of got a lot more involved with the response supporting ICU team so a lot of our time just kind of went into pandemic mode so I never really got the opportunity to discuss it fully with my team members.”

P12, Pharmacist.

#### Education & training

All participants identified shortfalls in their professional training and/or education they undertook and proposed how different aspects could be improved. Some have also thought about making changes to the way they will provide training in the future. There was emphasis on changing pharmacy education to be more patient/communication focused. This involves allowing students more exposure to patients in healthcare settings during their undergraduate degree, to develop communication skills required to provide adequate patient care in practice. Some nurses also indicated that doing a role reversal simulation experience like ‘A Life in a Day’ promotes further learning and is a more effective method for teaching than traditional methods.

“I think something similar should happen with other cases, breast cancer, colorectal,… it does make you learn and go away and read around the topic if you’re not 100% on that topic.”

P17, Nurse.

“In undergraduate courses you learn about all the drugs and how to counsel patients, but I don’t suppose you really learn so many skills about talking to people. … it’s really hard to learn… but it’s like an injection of experience that you get through life in a day I think.”

P4, Pharmacist.

“I am in the middle of overhauling a training package, my big thing is to try and incorporate the patient experience into that… To try and get them to put themselves in that patient position or the relative…”.

P12, Pharmacist.

## Discussion

This is the first study to evaluate the experience and impact of a RRS on oncology HCPs. Usually, simulation education in the field of healthcare is used to obtain the skills of a future professional or to teach skills to be attained for further specialisation and enhanced practice. Rarely is simulation used to explore the vulnerability of a patient receiving care. Through props and role play, the ‘A Life in a Day’ simulation experience incorporates various dimensions and senses; touch, sight, hearing, feelings, and one’s imagination. This has rarely been done. The experience is designed in such a way that participants carry out their usual daily activities, at home, work or elsewhere and are not restricted to a simulation centre or teaching environment. This is a different feature that many simulation methods have not utilised. Thus, this study contributes to the growing evidence that supports the use of RRS.

Stimuli within the experience induced an emotional response from participants and as a result, participants were able to relate with the feelings of patients that go through their cancer journeys. This was effective in helping participants have a better holistic understanding of their patients. The data suggests a strong link between the increase in empathy for patients and the increased knowledge of cancer patients’ journeys. This is important to note as it led to reflection on practice and the plan to change practice. This finding is supported by other studies utilising RRS. An immersive simulation qualitative study, from Queen’s University, Belfast [14], involved ten 4th year medical students who simulated being a patient with melanoma by wearing a mole tattoo and carrying out various tasks during the day. Some of the themes that emerged were; transformative introduction to patienthood and seeing cancer patients in a new light. Within these themes, students mentioned having a better understanding of the anxieties and struggles cancer patients face and having more empathy for them after the experience [14].

The simulation experience encouraged participants to reflect and share their thoughts about their current practice and attitudes. This was vital in motivating changes within practice. Results show that impacts from the experience generally translated into changes in practice, which ranged from changing consultation styles to taking on more responsibility in their roles. Pharmacy participants were inspired to be more holistic and ‘present’ in patient care, whereas registered nurses felt that they were often already incorporating these aspects into their clinical practice. Instead, more nurses felt that the simulation experience prompted them to refine their knowledge on the disease state and symptoms. These differences in impact highlight different needs within the respective practices, which is also evident in ‘A qualitative study exploring how pharmacist and nurse independent prescribers make clinical decisions’ [15]. Pharmacists were generally less interactive with patients compared to nurses and expressed their lack of confidence to perform physical examinations [15]. Pharmacists relied on reports and medical notes to aid their decision making, whereas more nurses directly involved patients in their decision making [15].

As some cohorts carried out the simulation experience during the Covid-19 pandemic, participants were faced with more telephone and virtual consultations than usual. This emphasised the importance of effective communication skills. Some participants found it challenging to show empathy through telephone consultations, especially if rapport had not been previously established. As telephone consultations have been demonstrated to be effective communication methods within healthcare [16], they will be incorporated into healthcare practice going forward. For this reason, it is extremely important that HCPs are adequately trained to continue providing holistic and patient centred care, even via telecommunication.

Participants were eager to share and promote the experience which showed the acceptance and approval of ‘A Life in a Day’ among participants. Although expectations for changes in practice were based on direct patient care, the experience also inspired participants to make changes in their approach to the training and education of others. This is an equally important aspect to consider when providing holistic care. Most participants suggested that education, especially in the pharmacy undergraduate program, would benefit from more patient facing elements. Giving students the opportunity to practice soft skills (such as problem solving, communication, teamwork, interpersonal skills, professional attitude, work ethic) through simulation may facilitate a smoother transition into practice. As these skills are usually attained through extensive experience, the use of simulation can equip students for a holistic approach to patient care early on in practice. As suggested by participants, simulation education can also be applicable to the continued professional development (CPD) of pharmacists, registered nurses and other HCPs.

### Limitations

The Covid-19 pandemic meant that many HCPs were occupied with work commitments which limited the response rate to interview invitations. A few participants that took part in the experience were experienced in their fields; some were clinical nurse specialists (CNSs) and some pharmacists were either managers of departments or specialists in the field of oncology. This meant that feedback and reflections were slightly biased to more experienced HCPs. Elements learnt from ‘A Life in a Day’ were sometimes already gained through extensive experience in practice which may have contributed to some not making changes to their practice. The conclusions of impact for this study cannot be extrapolated to that of a different cohort of participants with different levels of experience, job roles and education levels.

## Conclusion

This experience demonstrated that the impacts of ‘A Life in a Day’ go beyond increasing empathy to encourage practical change. RRS such as ‘A Life in a day’, is an exciting venture with huge potential which should be further explored in different disease states and amongst other HCPs within the MDT. Additionally, at professional practice level, it is recommended that simulation is used as a method of CPD for registered nurses, pharmacists and other HCPs and should be included in the curricula of undergraduate courses of all HCPs.

## Data Availability

The datasets used and/or analysed during the current study are available from the corresponding author on reasonable request.

## Abbreviations

App: application
BOPA: British oncology pharmacy association
CNS: clinical nurse specialists
CPD: continued professional development
HCP: healthcare professional
KUREC: Kingston University research ethics committee
MDT: multidisciplinary team
OSCE: objective structured clinical examination
RCC: renal cell carcinoma
RRS: role reversal simulation
UKONS: UK oncology nursing society
U.S.: United States
VH: virtual human
